# Impact of artificial intelligence-based and traditional image preprocessing and resampling on MRI-based radiomics for classification of papillary thyroid carcinoma

**DOI:** 10.1093/bjrai/ubaf006

**Published:** 2025-04-15

**Authors:** Abdalla Ibrahim, Ramesh Paudyal, Akash Shah, Nora Katabi, Vaios Hatzoglou, Binsheng Zhao, Richard J Wong, Ashok R Shaha, R Michael Tuttle, Lawrence H Schwartz, Amita Shukla-Dave, Aditya Apte

**Affiliations:** Department of Radiology, Memorial Sloan Kettering Cancer Center, New York, 10065 NY, United States; Department of Medical Physics, Memorial Sloan Kettering Cancer Center, New York, 10065 NY, United States; Department of Radiology, Memorial Sloan Kettering Cancer Center, New York, 10065 NY, United States; Department of Pathology, Memorial Sloan Kettering Cancer Center, New York, 10065 NY, United States; Department of Radiology, Memorial Sloan Kettering Cancer Center, New York, 10065 NY, United States; Department of Radiology, Memorial Sloan Kettering Cancer Center, New York, 10065 NY, United States; Department of Surgery, Memorial Sloan Kettering Cancer Center, New York, 10065 NY, United States; Department of Surgery, Memorial Sloan Kettering Cancer Center, New York, 10065 NY, United States; Department of Medicine, Memorial Sloan Kettering Cancer Center, New York, 10065 NY, United States; Department of Radiology, Memorial Sloan Kettering Cancer Center, New York, 10065 NY, United States; Department of Radiology, Memorial Sloan Kettering Cancer Center, New York, 10065 NY, United States; Department of Medical Physics, Memorial Sloan Kettering Cancer Center, New York, 10065 NY, United States; Department of Medical Physics, Memorial Sloan Kettering Cancer Center, New York, 10065 NY, United States

**Keywords:** MR radiomics, image preprocessing, thyroid carcinoma, image resampling

## Abstract

**Objectives:**

This study aims to evaluate the impact of image preprocessing methods, including traditional and artificial intelligence (AI)-based techniques, on the performance of MRI-based radiomics for predicting tumour aggressiveness in papillary thyroid carcinoma (PTC).

**Methods:**

We retrospectively analysed MRI data from 69 patients with PTC, acquired between January 2011 and April 2023, alongside corresponding histopathology. MRI scans underwent N4 bias field correction and resampling using 10 traditional methods and an AI-based technique, synthetic multi-orientation resolution enhancement (SMORE). Radiomic features were extracted from the original and preprocessed images. Recursive feature elimination with random forests was used for feature selection, and predictive models were developed using XGBoost. The performance of the model was assessed by calculating the area under the receiver operating characteristic curve (AUC) across 1000 iterations.

**Results:**

The combination of the correction of the bias field of N4 with SMORE resampling produced the highest mean AUC (0.75), significantly outperforming all traditional resampling methods (P<.001). The lowest mean AUC (0.66) was observed with nearest neighbour resampling. Texture-based radiomic features, particularly the standard deviation of the grey-level co-occurrence matrix autocorrelation, were frequently selected in models using SMORE-resampled images.

**Conclusions:**

Preprocessing techniques critically influence the predictive performance of MRI-based radiomics in PTC. The combination of N4 bias field correction and SMORE resampling enhances accuracy, highlighting the necessity of optimizing preprocessing pipelines.

**Advances in knowledge:**

This study demonstrates the superiority of AI-driven preprocessing techniques, such as SMORE, in improving MRI radiomic models, paving the way for enhanced clinical decision-making in PTC management.

## Introduction

Radiomics is a data-centric field involving the high-throughput extraction of quantitative features from medical images,^1^ including computed tomography^2^^,^^3^ and magnetic resonance imaging (MRI).^4^ These image features capture the shape, histogram, and textural and higher order statistics features from a specified region of interest (ROI) based on the pixel’s spatial distribution of signal intensities,^5^^,^^6^ which are typically not perceptible to human eyes. Despite the rapid growth of radiomic studies, the lack of standardization and test-retest studies have hampered its translation into radiological practice.^7^^,^^8^ Recently, an International Image Biomarker standardization Initiative (IBSI) recommended the use of a set of standardized image features, which enabled verification and calibration across different software.^9^

A prerequisite for image feature extraction from qualitative anatomical MR images is the preprocessing step, including the normalization of signal intensities, which are represented in arbitrary units and slightly differ between patients, introducing variations in the contrast, as they are highly dependent on MRI data acquisition, scanner field strength, and the image reconstruction method.^10^ MR images may also suffer from geometric distortion, ghosting, and susceptibility artefacts depending on the organ of study.^11^^,^^12^ These factors can lead to measurement biases and inconsistencies in image feature values.^13^^,^^14^ To account for these inconsistencies, normalization methods have been applied to MR images, including N4 bias field correction, which reduces the inhomogeneity within tissue, and *Z*-Score, which scales each feature to have 0 mean and a variance of 1.^15–18^ In addition, image resampling to a unified voxel volume has been investigated in radiomics analyses.^19^^,^^20^ Traditional resampling methods, including linear, nearest neighbour, B-spline, Gaussian, and windowed sinc, are readily available across various image processing software platforms.^21^ Most recently, Zhao et al developed a novel synthetic multi-orientation resolution enhancement (SMORE) method using convolutional neural networks (CNNs) that restores image quality by improving resolution and reducing aliasing in MR images.^22^ SMORE method has been shown to be visually and quantitatively superior to previously reported methods.

Radiomics has promised to improve clinical decision-making, including for patients with cancer.^23–25^ In particular, radiomics and machine learning (ML) approaches have been increasingly applied to different imaging modalities for thyroid cancer.^26–29^ For instance, Hu et al evaluated the utility of image features obtained from multi-contrast MR images (T2weighted (w), contrast-enhanced (CE)-T1w, and diffusion-weighted (DW)) for the detection of lymph node metastases (LNM) in papillary thyroid cancer (PTC),^30^ while Dai et al predicted tumour aggressiveness using CE-T1w, T2w, DW images.^31^ Wei et al utilized CE-T1w, T2w, and DW images to predict extrathyroidal extension.^32^ Although these studies made valuable contributions, they did not incorporate data preprocessing steps in their methodologies. In contrast, Wang et al performed N4 bias field correction and image intensity rescale prior to feature extraction and reported that ML-based multiparametric MR (T2w and CE-T1w) images and apparent diffusion coefficient map radiomics could accurately distinguish aggressive from non-aggressive PTC preoperatively.^29^ These studies form a foundation for further radiomic investigations in PTC.

PTC has various subtypes, each exhibiting distinct histological, imaging and prognostic characteristics.^33^ The presence of local invasion, extra-glandular invasion, LNM or distant organ metastasis defines invasive thyroid cancer.^33^^,^^34^ Surgical resection remains the primary treatment for most aggressive thyroid cancers, often with poor prognoses.^35^ Despite imaging advances, pathological tissue examination remains the gold standard for diagnosis. Hence, early diagnosis and characterization of thyroid cancer aggressiveness holds paramount importance. However, its efficacy is influenced by various factors, including nodule size and location, presence of calcification and liquefaction.^36^

There is an unmet need to expand the use of the radiomics feature with the ML approach and explore its implications for improving risk stratification in PTC. To the best of our knowledge, this study is the first to investigate the potential of an MR radiomics pipeline that consists of several image preprocessing and harmonization steps ahead of feature extraction for PTC. We assessed the impact of 10 traditional image resampling techniques and Artificial Intelligence (AI)-based SMORE sampling on the performance of radiomics signatures. Traditional resampling techniques included Nearest neighbour, linear, Gaussian, label Gaussian, Hamming windowed sinc, cosine windowed sinc, Welch windowed Sinc, Lanczos windowed sinc, and Blackman windowed sinc, available in SimpleITK.^37^ This study aims to investigate the impact of the standardization of MR images using traditional and AI preprocessing methods that are important towards developing novel radiomics-based ML models to predict tumour aggressiveness of PTC.

## Methods

### Patient data

The present study retrospectively collected 71 patients (*n* = 71) evaluated for thyroidectomy who were enrolled after thyroid nodule FNA demonstrated either PTC or suspicion of thyroid cancer and had preoperative MRI exams between January 2011 and April 2023. Inclusion criteria required that patients have both MRI imaging and tissue pathology results. Baseline T2w images were analysed in this study, rather than multiparametric images, to identify the best combination of preprocessing and standardization methods for radiomics analysis. The study was approved by our institutional review board.

### MR data acquisition

MRI exams were performed on a 3.0 T MR scanner (Discovery MR 750, GE Healthcare, Milwaukee, WI) and 1.5 T (Optima MR450w, GE Healthcare, Milwaukee, WI) with a neurovascular phased‐array coil before surgery. T2w, fat-suppressed acquisition (2D multislice) images were acquired with the following MR acquisition parameters: repetition time/echo time, TR/TE = 2500-5066 ms/77-124 ms, slice thickness = 5 mm, and FOV = 2024 mm covering the entire thyroid gland (25-30 slices).

### Histopathology

**Figure 1.**
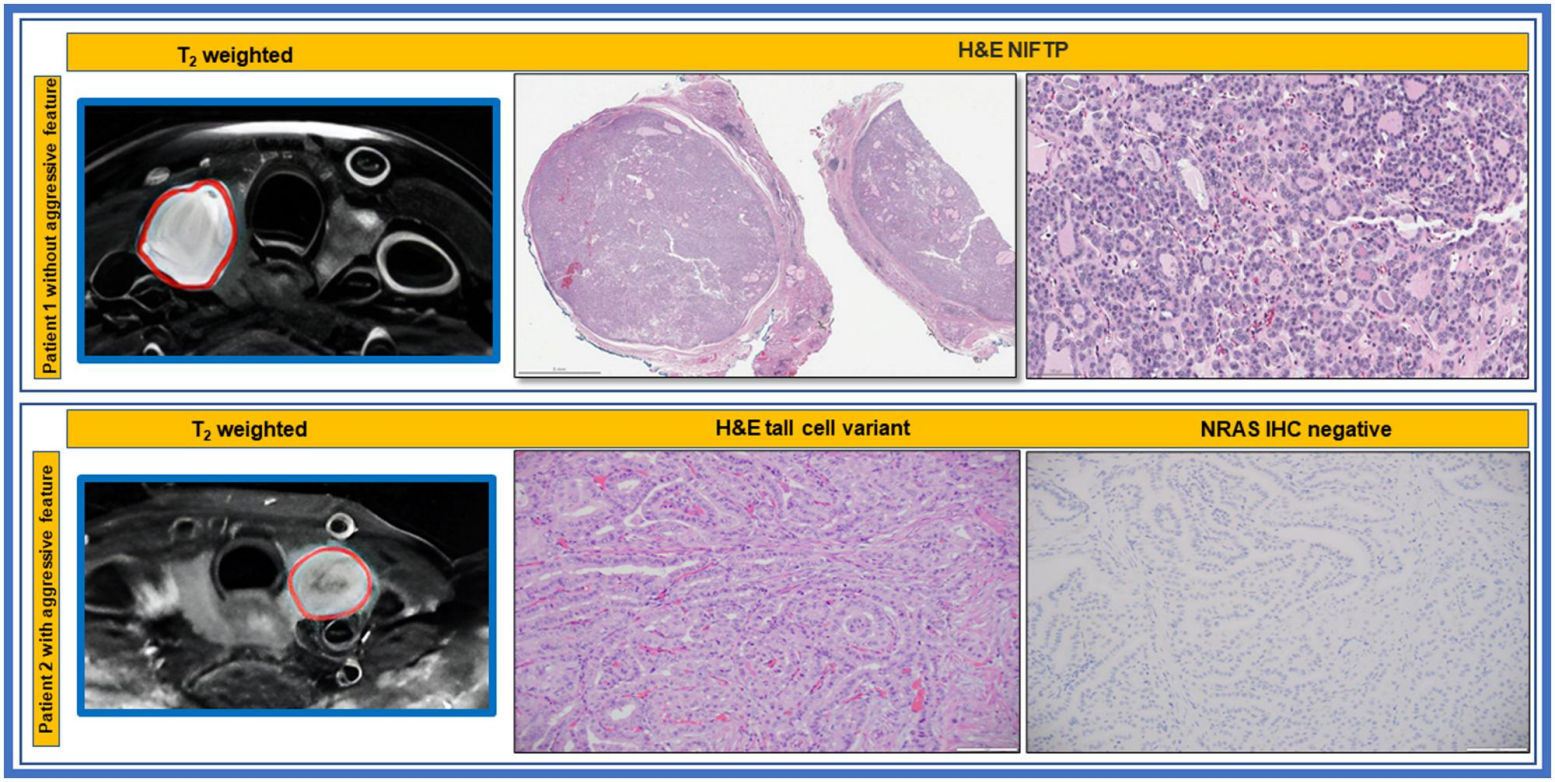
T2 weighted MR and histopathologic images of patients with and without aggressive tumour features. Top Row: The H&E shows NIFTP, noninvasive follicular thyroid neoplasm with papillary-like nuclear features, showing noninvasive well circumscribed borders and follicular growth pattern. Bottom Row: The H&E shows papillary thyroid carcinoma, tall cell variant as well as the negative IHC for oncogenic NRASQ61R, an activating mutation of RAS oncogene.

Pathology details have been described previously by Lu et al.^38^ Surgical specimens of the PTC were taken after radical thyroidectomy or lobectomy under the supervision of a pathologist with more than 10 years of experience. Paraffin-embedded tissue blocks were obtained for each surgically resected specimen by sectioning each tumour and staining the sections with haematoxylin and eosin. The pathologist evaluated tumour aggressiveness using 6 established histopathologic criteria, including tall cell subtype, extrathyroidal extension (ETE), vascular or capsular invasion, necrosis, distant metastases, and regional metastases.^39^ The presence of any one of these features in tumours was deemed aggressive. Figure 1 shows an example of aggressive and non-aggressive histopathology images.

### Image preprocessing and analysis pipeline

The tumours were segmented on T2w images by neuroradiologists with over ten years of experience.^40^ As patient data were prospectively collected, acquisition parameters varied among each patient’s scans, potentially influencing radiomic feature values. Given the lack of standardization in MRI intensity values across T2w scans, multiple preprocessing steps were required to ensure comparability between patients, as described below.

**Figure 2.**
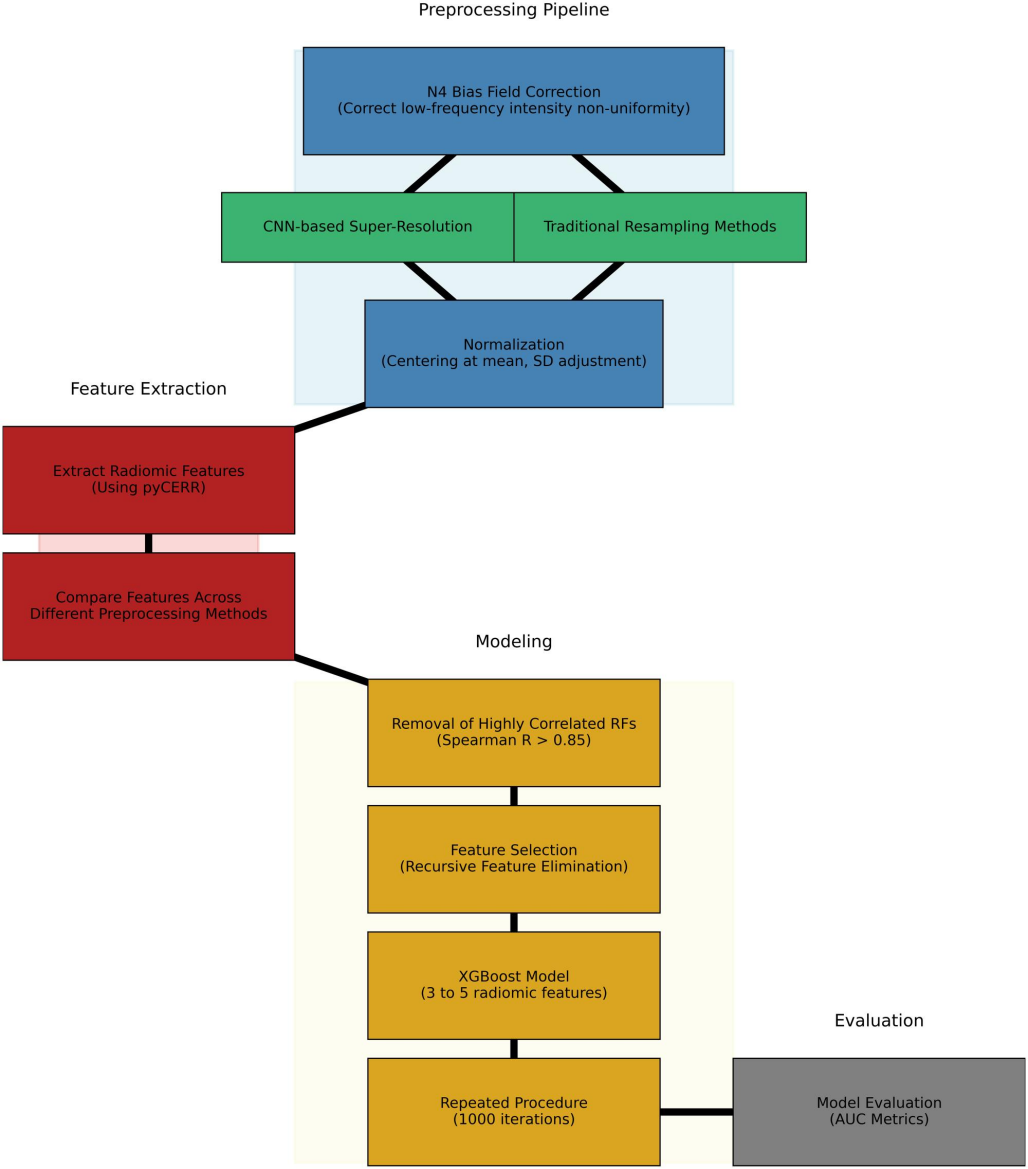
Workflow for preprocessing MR image pipeline.

The pipeline (Figure 2) begins with N4 bias field correction to mitigate low-frequency intensity non-uniformity in the MRI data, as this bias can distort the overall signal intensity.^41^ Images were resampled using traditional techniques available in SimpleITK as well as a CNN-based super-resolution algorithm to achieve isotropic scans based on the original in-plane resolution of each scan.^22^ The SMORE algorithm was implemented using the published self-supervised version without any modifications. CNN-based resampled images were further linearly resampled to a fixed isotropic resolution to ensure the same resolution across all patient scans. IBSI-compatible pyCERR software (github.com/cerr/pycerr) was used to extract radiomics features.^42^

**Figure 3.**
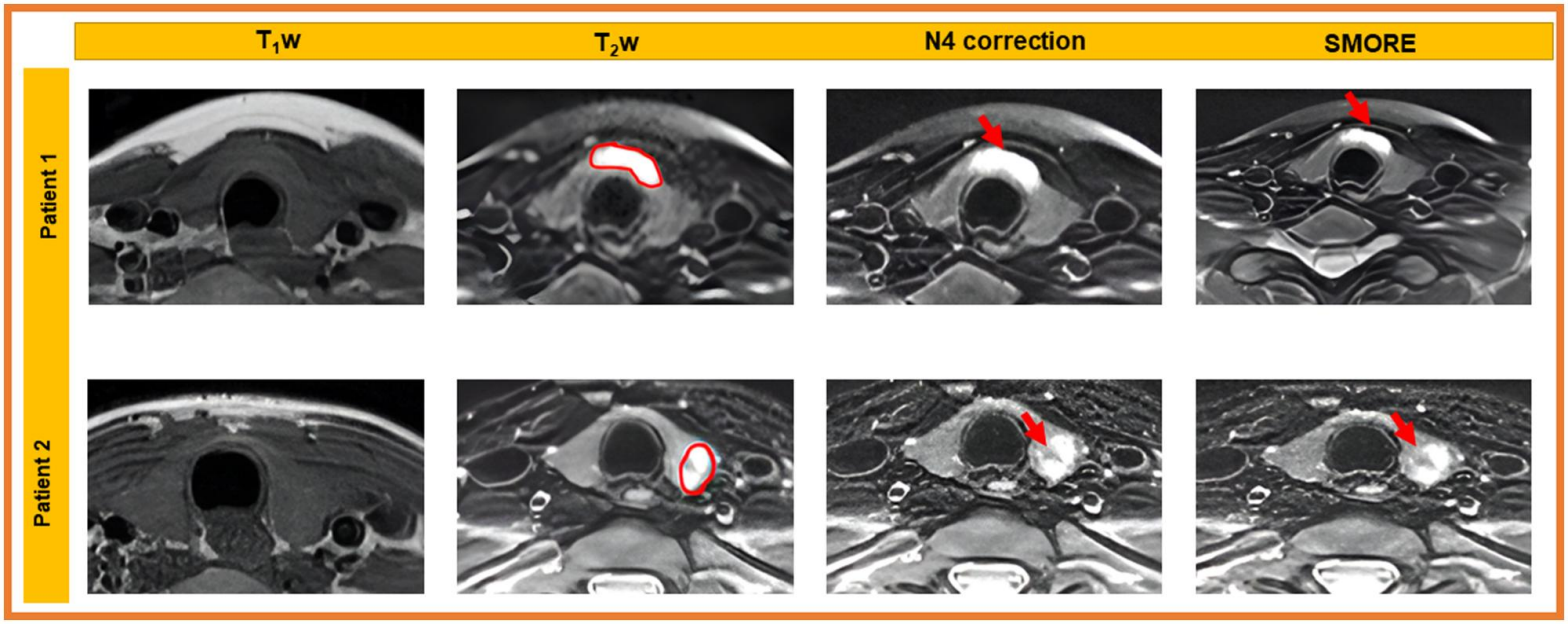
T1w images and fat-suppressed T2-weighted images for 2 patients demonstrate STIR hyperintense tumour in the thyroid isthmus (patient 1, ROI) and T2 heterogeneous tumour in the right thyroid lower pole (patient 2, ROI). Image quality for both patients is maintained between N4 bias-field corrected images and SMORE images, noting tumour visibility without significant artefacts.

After N4-bias field correction of image intensities, the images were standardized to robust *Z*-scores using median and mean absolute deviation of intensities within respective regions of interest. Robust *Z*-score = 0.6745*(xi − $\tilde{\text{x}}$)/MAD; where xi is voxel intensity, $\tilde{\text{x}}$ and MAD are the median and mean absolute deviation of voxel intensities within the tumour ROI. Figure 3 shows examples of the SMORE MR scans.

Radiomics features included first-order statistics and higher-order textures based on grey-level co-occurrence matrix (GLCM), Run Length matrix (RLM), Size Zone matrix (SZM), neighbourhood grey Level dependence matrix (NGLDM), and neighbourhood grey Tone difference matrix (NGTDM). To calculate texture features, the images were discretized using a bin width of 0.05 with 1st bin corresponding to the *Z*-score of −5. A voxel offset of 1 was used for GLCM features, and a patch size of 3 × 3×3 was used for NGLDM and NGTDM texture features.

To assess the efficacy of the preprocessing pipeline, radiomic features were extracted from 3 image types: original T2w images, N4-bias field-corrected T2w images, and N4-bias field-corrected T2w images resampled using ten traditional resampling methods using Simple ITK Python library (Nearest neighbour, Linear, Gaussian, label Gaussian, Hamming windowed sinc, cosine windowed sinc, Welch windowed sinc, Lanczos windowed sinc, and Blackman windowed sinc) and AI-based SMORE resampling. The radiomic features generated from each of these image types were used separately to model PTC aggressiveness.

### Statistical analysis

Following the preprocessing steps, patients were randomly split into 75% training and 25% testing cohorts. Feature selection was performed using Recursive Feature Elimination (RFE) with Random Forests,^43^ followed by hyperparameter tuning using RandomizedSearchCV to optimize model performance,^44^ which included 5-fold cross validation. Top 5 features were retained in the final model for each experiment. Performance was measured using the area under the receiver operating characteristic curve (AUC) statistics on the testing cohort. The process was repeated 1000 times to ensure robust results and mitigate overfitting.

In addition, we compared AUC values from the different models using paired t-tests or Wilcoxon signed-rank tests, depending on the normality of the data, to determine the optimal preprocessing technique.^45^ Normality was checked using the Shapiro-Wilk test. These comparisons were used to select the most effective resampling method and preprocessing approach for subsequent analysis. All statistical analyses were conducted using Python, leveraging XGBoost (an open-source software library which provides a regularizing gradient boosting framework) for model building.^46^

## Results

### Patient characteristics

Out of 71 patients, 2 patients were further excluded in this study. One patient had susceptibility artefact in the thyroid lobe; therefore, no tumour was visible to delineate, and the other patient had chronic lymphocytic thyroiditis. For this latter patient, only features suggesting thyroiditis were seen on T2w images and the tumour was not visible. The remaining 69 patients were included in further analyses. The median patient age was 43 years, ranging between 19 and 71 years. Overall, 43 (62.3%) patients had tumours with aggressive features (Table 1). The neuroradiologists reviewed standard anatomical T1w images, and tumours were visible in only 32% of these patients. The standardization pipeline was applied to T2w images.

**Table 1.** Patient characteristics.

Characteristic	*n* (%)
Age at diagnosis, years (median)	43 (19-71)
Gender (male)	23 (33.3%)
**Aggressive features based on pathology**	
Tall cell	25 (36.76%)
Extrathyroidal extension	27 (39.71%)
Necrosis	0 (0%)
Vascular and/or tumour capsular invasion	10 (14.71%)
Regional metastases	30 (44.12%)
Distant metastases	0 (0%)

### AUC performance across preprocessing methods

**Figure 4.**
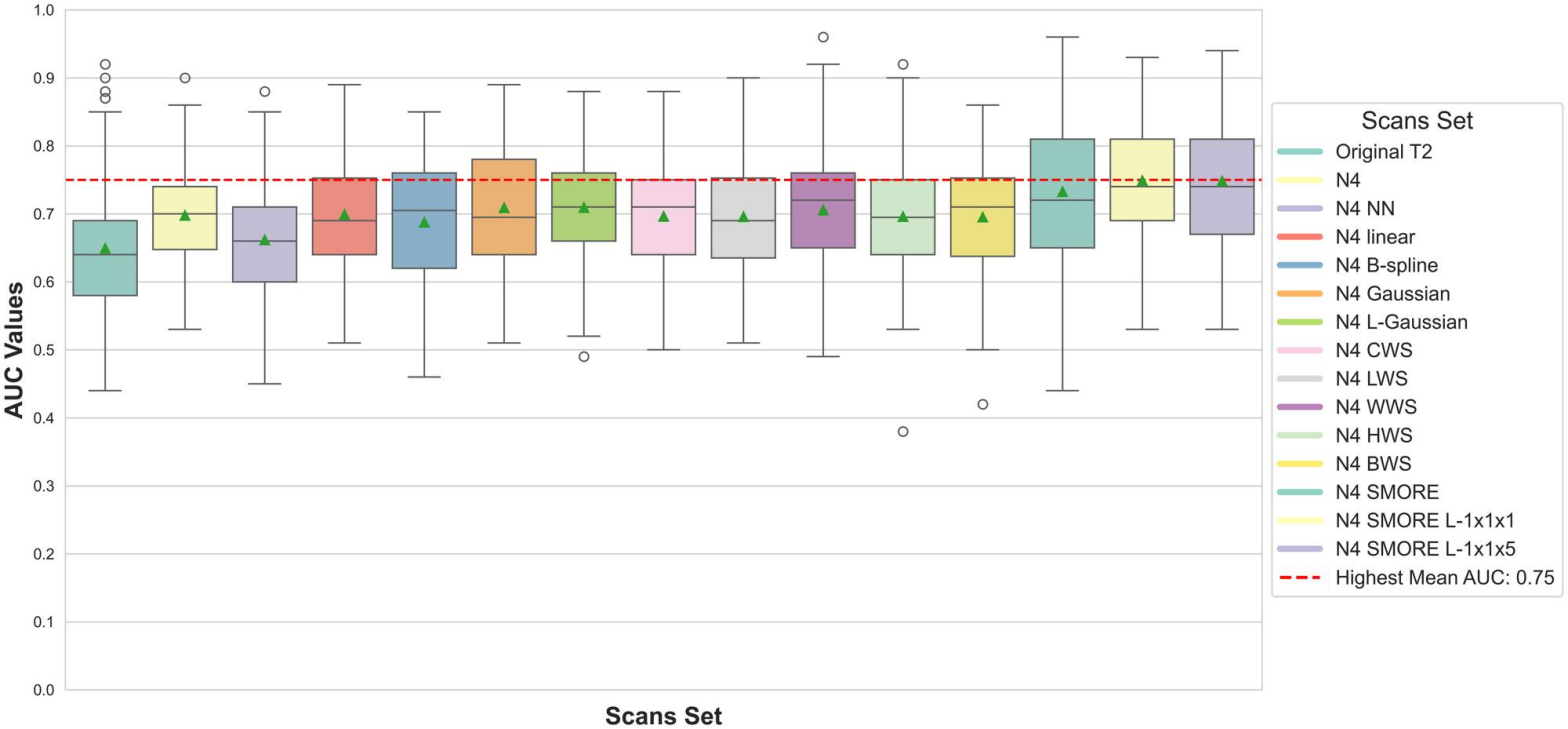
Box-and-whisker plots of AUC values for predicting the aggressiveness of PTC using the different sets of sampling methods. The red line represents the highest mean AUC across the scan sets.

The performance of radiomic signatures across the different preprocessing techniques was assessed using AUC on the testing cohorts. Figure 4 presents the AUC values from the various preprocessing pipelines across the runs. The highest-performing preprocessing techniques included N4 bias field correction with SMORE resampling, where the SMORE L-1x1x1 and SMORE L-1x1x5 scan sets achieved the highest mean AUC of 0.75. In contrast, the lowest performance was seen with N4 bias field correction followed by nearest-neighbour (NN) resampling, yielding a mean AUC of 0.66.

### Comparison of resampling techniques

**Figure 5.**
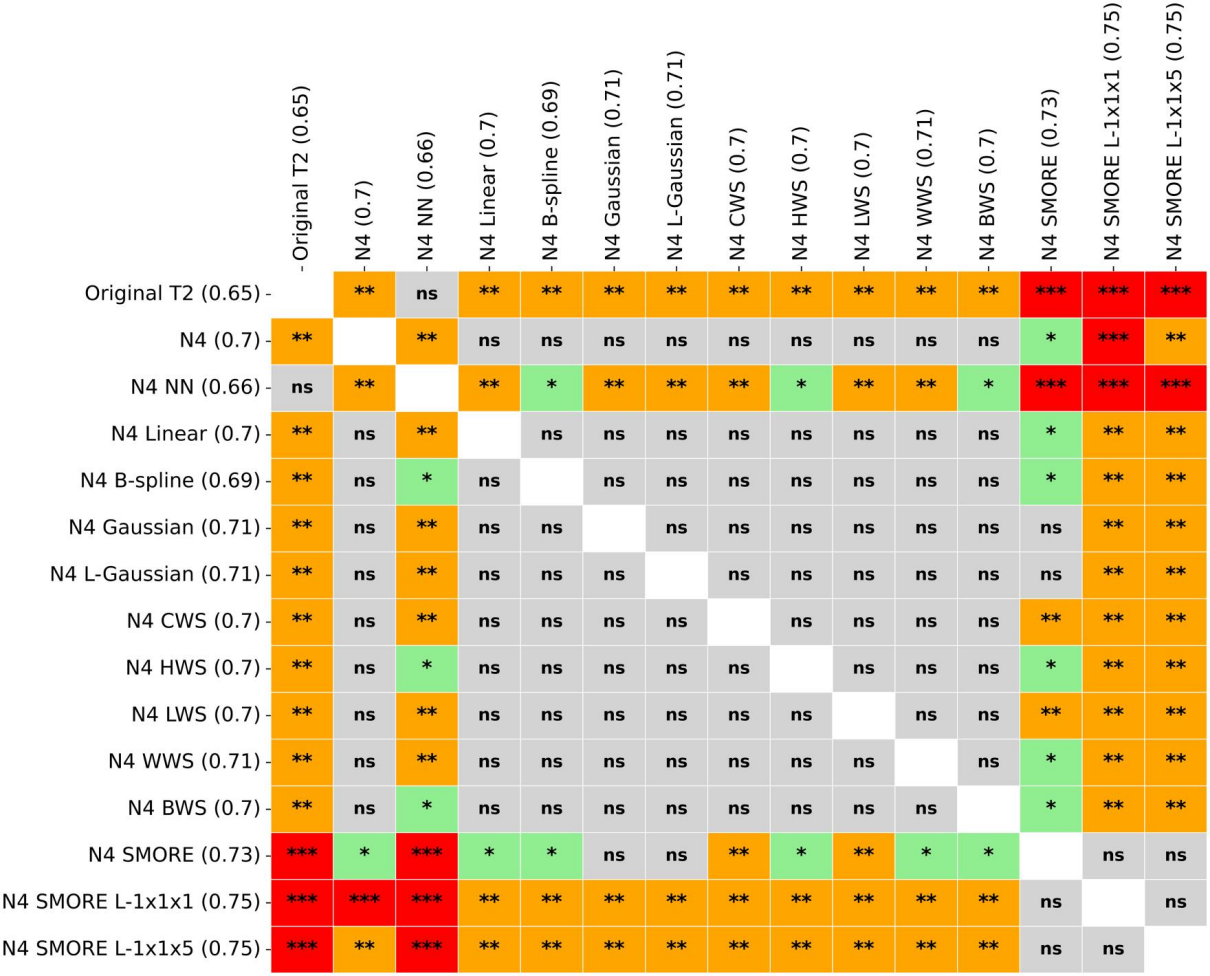
Statistical differences in AUC values across the sets of scans. ns = not significant; *= *P*<.05; **= *P*<.01; ***= *P*<.00001.

Wilcoxon signed-rank tests and paired t-tests were used to assess performance differences between each pair of preprocessing methods. Notably, N4 SMORE L-1x1x5 significantly outperformed N4 NN (P<1e−9) and other traditional resampling methods, such as B-spline and linear (P<.001). A paired t-test revealed significant improvements in AUC when using advanced techniques such as SMORE compared to traditional methods like Gaussian (*P* = .003) and original T2w images (P<1e−6). In contrast, no significant difference was observed between N4 SMORE and N4 SMORE L-1x1x1, indicating that variations in resolutions of SMORE sampling performed similarly well. Figure 5 summarizes the results of these comparisons, with significant *P*-values indicating where one preprocessing method outperformed another.

### Selected features across preprocessing methods

**Figure 6.**
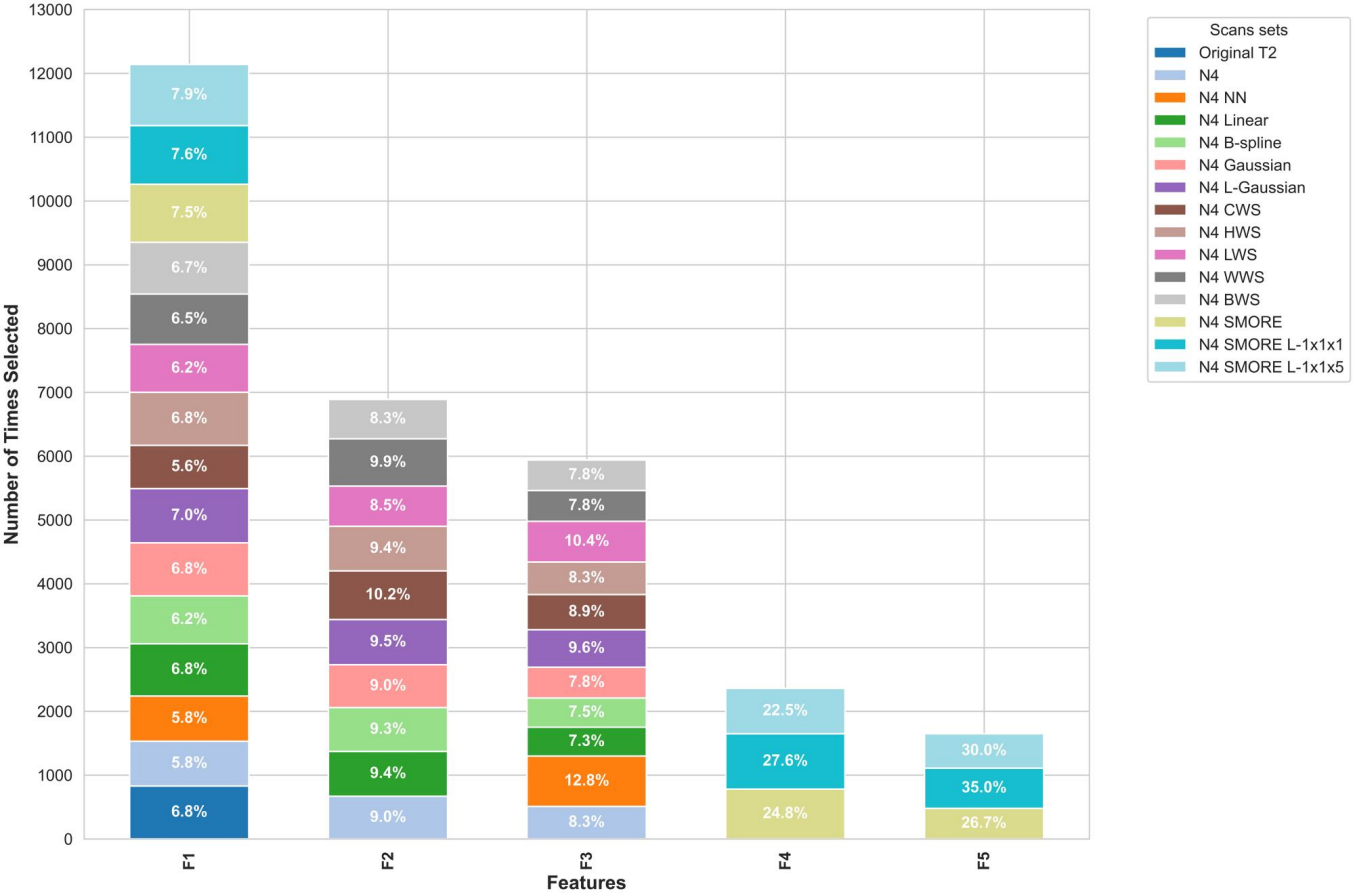
Most selected radiomic features F1: GLCM autoCorr, F2: GLCM haralick Corr, F3: GTDM Busyness, F4: GLCM jointMax, F5: GLRLM GrayLevel variance. F4 and F5 were almost unique to the models of SMORE scan sets.

We also examined the most frequently selected features across different preprocessing pipelines. Figure 6 highlights the features most commonly selected by RFE across the runs. The most frequently selected feature was the standard deviation of GLCM autoCorr across different directions, which was chosen 12140/15000 times (80.9%) across all pipelines, with particularly high selection in models using N4 SMORE L-1x1x5 (960 times) and N4 SMORE L-1x1x1 (920 times). Other top features included the standard deviation of GLCM haralickCorr across all directions and GTDM Busyness. The features standard deviation of GLCM jointMax across all directions and GLRLM GrayLevel Variance were predominantly selected in models that used SMORE-based methods, emphasizing the potential value of second-order texture features in predicting PTC aggressiveness.

Several features, such as the standard deviation of GLCM invar across all directions and Shape Compactness1, also showed high selection consistency across different preprocessing methods, indicating their robustness in various pipelines. Interestingly, some features like GLCM jointMax and GLRLM short Run Emphasis were more frequently selected in advanced resampling methods, suggesting that specific preprocessing techniques can enhance feature relevance in prediction models.

## Discussion

In this study, we investigated the influence of traditional and AI-based image preprocessing and resampling techniques on the performance of MRI-based radiomic features in predicting PTC aggressiveness. Results revealed that the highest average performance was achieved by scans preprocessed with N4 bias field correction combined with SMORE resampling with unification of voxel size across patients. These approaches outperformed other resampling techniques, including traditional methods like Gaussian resampling and linear resampling, in terms of AUC performance. Nearest neighbour resampling yielded the lowest performance among the tested techniques.

The superior performance of SMORE-based resampling can be attributed to its ability to enhance spatial resolution and mitigate aliasing artefacts, resulting in improved radiomic feature extraction.^22^ N4 bias field correction likely contributed to this improvement by correcting for low-frequency intensity non-uniformity, enhancing the signal-to-noise ratio, and improving the discernibility of radiomic features.^41^

Notably, texture-based features such as GLCM autoCorr emerged as the most consistently selected feature across multiple preprocessing methods. This feature, which captures the autocorrelation of grey-level co-occurrence matrices, played a crucial role in the predictive models, demonstrating its potential in the classification of PTC aggressiveness.^47^ The prominence of second-order texture features, particularly those derived from the GLCM matrix, suggests that SMORE resampling enhances the extraction of these higher-order statistical measures, potentially improving model performance compared to traditional resampling techniques.

Interestingly, first-order features, which were more commonly selected in the original and N4 bias-corrected scans, were less frequently selected following SMORE resampling. This observation suggests that SMORE may improve the capture of textural characteristics, while first-order statistics may become less relevant in these enhanced images. Additionally, the variability in intensity values introduced by SMORE resampling might affect the relevance of certain first-order features, which rely heavily on image intensity values.^40^

Among the most frequently selected features were GLCM haralickCorr and GTDM Busyness. These features have previously been associated with the quantification of tumour heterogeneity, and their consistent selection across different resampling pipelines highlights their relevance in PTC aggressiveness prediction.^1^ The ability of these features to reflect the structural complexity and heterogeneity of tumours further underscores the value of texture analysis in radiomic studies.

Our findings suggest that while SMORE resampling outperformed other methods, bias field correction alone also yielded strong predictive performance, highlighting the importance of addressing MRI intensity non-uniformity 16. The improvement with SMORE resampling may be due to the increase in in-plane resolution to 1 × 1 mm and the enhancement of the *z*-axis resolution from 5-6 to 1 mm. However, the interpolation required to add slices between the originals may introduce noise and reduce the predictive capability of some radiomic features. This aligns with previous research, which has suggested that image resampling may not always improve radiomic model performance.^48^

Our results underscore the potential of radiomic features in predicting the aggressiveness of PTC, offering valuable clinical insights that could enhance patient management. However, further research is essential to validate these findings, particularly through the use of external datasets, to enhance the generalizability of radiomic signatures in clinical practice.

Despite the rigorous statistical analysis employed in this study, a minor limitation should be acknowledged. The small sample size limits the generalizability of our results. Future studies should incorporate larger, multi-institutional datasets with standardized imaging protocols to improve the reliability and applicability of radiomic models in clinical settings.

## Conclusion

In conclusion, this study demonstrates that traditional and AI-based image preprocessing and resampling techniques impact the performance of radiomic features in predicting PTC aggressiveness from T2 MR images. AI-based SMORE resampling, particularly in combination with linear resampling, emerged as the optimal choice among the methods examined. These findings highlight the importance of selecting appropriate preprocessing pipelines in radiomic studies to emphasize the potential of radiomic features as powerful tools in the analysis of MRI in clinical decision-making for PTC.

## Data Availability

Data can be requested by contacting the corresponding author.
